# Comparison of central FLAIR hypointensity and central vein sign on FLAIR* in a diagnostic cohort

**DOI:** 10.1016/j.ejrad.2026.112707

**Published:** 2026-01-29

**Authors:** Karlo Toljan, Moein Amin, Lynn Daboul, Kunio Nakamura, Andrew J. Solomon, Nancy L. Sicotte, Russell T. Shinohara, Daniel S. Reich, Pascal Sati, Daniel Ontaneda

**Affiliations:** a Mellen Center for Multiple Sclerosis Treatment and Research, Cleveland Clinic, Cleveland, USA; b Translational Neuroradiology Section, National Institute of Neurological Disorders and Stroke, National Institutes of Health, Bethesda, MD, USA; c Cleveland Clinic Lerner College of Medicine, Cleveland, OH, USA; d Department of Neurology, Brigham and Women’s Hospital, MA, USA; e Department of Biomedical Engineering, Cleveland Clinic Research, Cleveland Clinic, Cleveland, USA; f Department of Neurological Sciences, Larner College of Medicine, University of Vermont, Burlington, VT, USA; g Department of Neurology, Cedars-Sinai Medical Center, Los Angeles, CA, USA; h Department of Biostatistics, Epidemiology, and Informatics, University of Pennsylvania Perelman School of Medicine, Philadelphia, PA, USA; i Penn Statistics in Imaging and Visualization Center, University of Pennsylvania Perelman School of Medicine, Philadelphia, PA, USA; j Center for AI and Data Science for Integrated Diagnostics, University of Pennsylvania Perelman School of Medicine, Philadelphia, PA, USA

**Keywords:** Biomarkers, Central vein sign, Multiple sclerosis, MRI

## Abstract

**Background:**

The central vein sign (CVS) is a neuroimaging biomarker in multiple sclerosis (MS) with high diagnostic specificity. CVS is best detected with high-quality susceptibility-sensitive MRI sequences. For concurrent detection of lesions and veins, FLAIR* was developed as a post-processing method to provide contrast for T2 hyperintense lesions (FLAIR) and paramagnetic hypointense veins (T2*-weighted). Occasionally, CVS-like features have been noted on FLAIR, but the reliability of this finding is unknown.

**Objective:**

To compare the central FLAIR hypointensity to FLAIR* CVS.

**Methods:**

Scans from the CentrAl Vein Sign in MS (CAVS-MS) pilot study were included for the analysis. A blinded rater assessed all lesions for CVS on 3-tesla post-contrast FLAIR*. A second blinded rater assessed the same lesions for central hypointensity on FLAIR images alone. Counts were compared between methods. The same approach was applied for a subset with available non-contrast FLAIR* lesion ratings.

**Results:**

With post-contrast FLAIR* CVS as the standard (n= 92; 1737 lesions), central FLAIR hypointensity demonstrated concordance of 64%, with sensitivity of 34% (95% CI, 30–37%) and specificity of 83% (95% CI, 81–85%). With non-contrast FLAIR* CVS as the standard (n= 38; 768 lesions), FLAIR demonstrated sensitivity of 40% (95% CI, 33–47%) and specificity of 85% (95% CI, 82–88%). Select 6 (≥6 central hypointense lesions) FLAIR was 59% accurate for a diagnosis of MS, with a lower specificity (63% vs. 90%, p= 0.008) in comparison to post-contrast FLAIR*.

**Conclusions:**

Assessment of CVS on FLAIR alone is unreliable and requires susceptibility-sensitive sequences to be clinically useful.

## 1. Introduction

The central vein sign (CVS) is a neuroimaging biomarker in multiple sclerosis (MS) reflecting perivenular demyelination on pathology [1,2]. When standard T2-weighted (T2w) sequences are combined with susceptibility-sensitive MRI techniques, it is possible to visualize both the central vein and the T2w hyperintense lesions characteristic for MS. Techniques such as the FLAIR* enable concurrent visualization of the T2w hyperintense lesions and veins employing 3D T2w FLAIR and T2*-weighted (T2*w) 3D echoplanar imaging [3]. Automated post-processing is needed to generate FLAIR*, which is comprised of coregistration, interpolation, and multiplication of FLAIR and T2*w images. This enables a highly suited contrast for the assessment of lesion CVS status according to North American Imaging in MS (NAIMS) Cooperative consensus [2], with post-contrast FLAIR* showing better performance for that purpose than non-contrast FLAIR* [4,5]. As a neuroimaging biomarker, CVS has substantial diagnostic value in MS, with the ≥ 40% threshold demonstrating a pooled sensitivity of 95% and specificity of 92% [6]. Similarly, abbreviated approaches with the presence of ≥ 3 CVS + lesions (Select 3) demonstrated a pooled sensitivity of 88%, while the approach with ≥ 6 CVS + lesions (Select 6) a pooled specificity of 89% [7]. Based on the diagnostic performance data, Select 6 was integrated into the MS criteria as part of the 2024 McDonald revisions [8].

There have been reports that central hypointensity can be seen in lesions on FLAIR images alone [9,10], with features resembling a central vein; this has raised the question of whether CVS can be characterized solely by FLAIR. If so, the diagnostic performance of central FLAIR hypointensity would be of wide interest in light of 2024 McDonald revisions incorporating CVS and overall technical feasibility. There are no formal studies investigating central FLAIR hypointensity as a diagnostic marker in MS. However, FLAIR is not optimized for demonstrating parenchymal venous structures, and without any dedicated studies assessing the value of this sporadic finding, its significance is unknown. This motivated an exploration of CVS rating based on FLAIR, followed by a comparison to FLAIR* as a standard. Data acquired as part of the CentrAl Vein Sign in MS (CAVS-MS) pilot study enabled a direct comparison [11].

## 2. Methods

### 2.1. Study design

Images obtained as part of the CAVS-MS pilot study were used for the analysis [11]. The CAVS-MS pilot study was a North American observational study designed to assess the feasibility of CVS acquisition across 10 participating academic centers. The study was approved by the Institutional Review Board at all included sites and consenting participants were enrolled. It was focused on the diagnostic performance of CVS based on FLAIR*, as one of the recommended approaches according to NAIMS consensus [2].

### 2.2. Participants

Participants of the CAVS-MS pilot study, aged 18–65 (inclusive), were referred to the participating academic site for a new clinical or radiological suspicion of MS, but without prior exposure to MS disease-modifying therapies. Of 97 recruited participants, 92 participants were considered for the main analysis, as 5 had inadequate imaging (artifacts in 4, and 1 missing post-Gd sequences). Diagnosis was determined by an adjudicating committee comprised of three MS experts (2 neurologists and 1 neurologist/neuroradiologist) using the McDonald 2017 criteria as the diagnostic standard. Thirty-seven were diagnosed with MS, 14 with clinically or radiologically isolated syndrome, and the rest with an alternative diagnosis [11].

### 2.3. MRI protocol

Images were acquired according to a standardized 3-tesla protocol (Supplementary table 1), as published previously [5,11]. Three sites used a Philips MRI scanner and 7 sites a Siemens MRI scanner. Imaging protocol included FLAIR (isotropic resolution, 1 mm) and high-resolution isotropic T2*-weighted segmented echo-planar imaging (EPI), with non-contrast and post-contrast acquisition [4,5,11–13]. FLAIR* was generated by automated postprocessing [3–5,11–13]. No additional post-acquisition standardization was done.

### 2.4. Lesion ratings

**Fig. 1.**
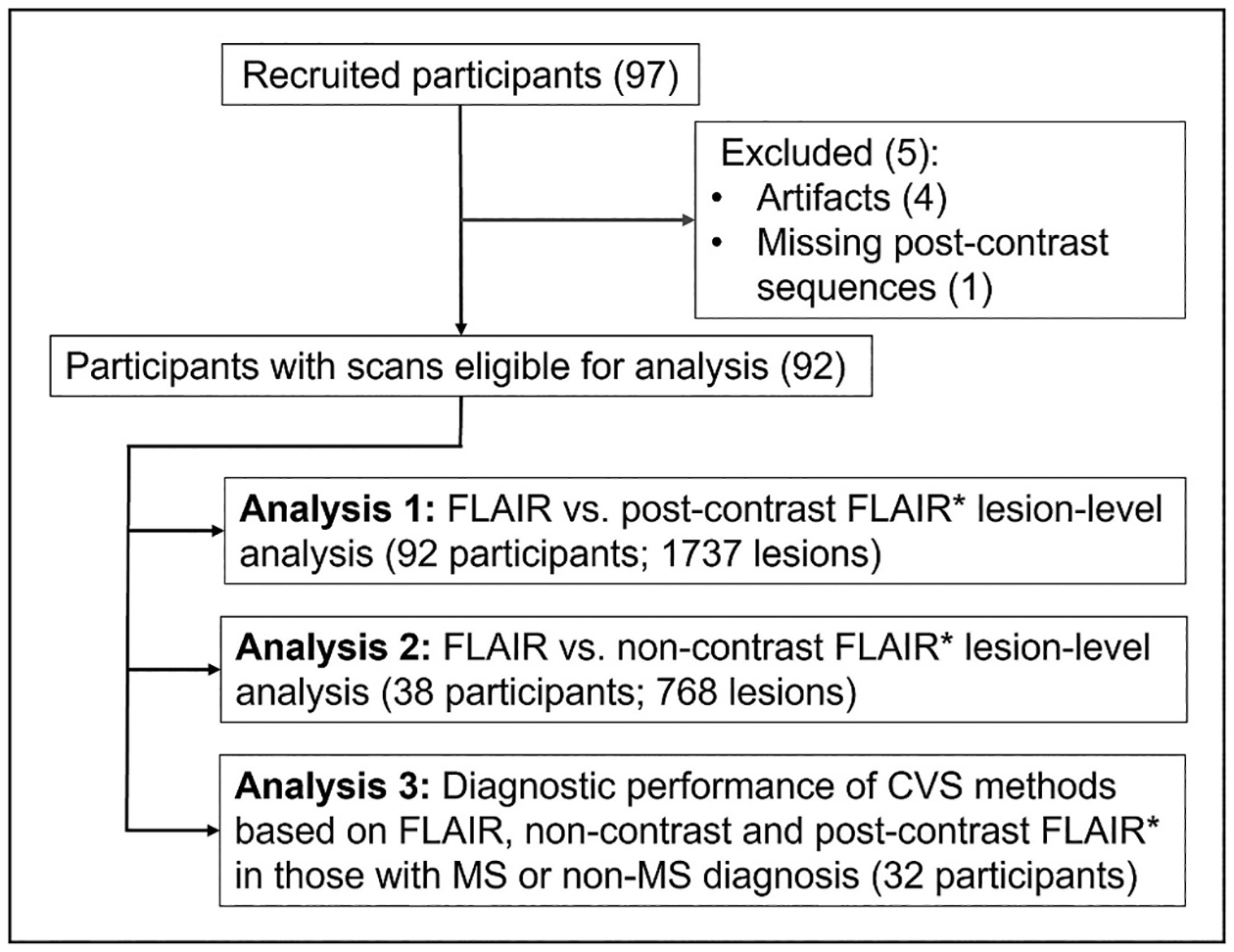
Flow diagram of study sample which was the basis for each respective analysis.

**Fig. 2.**
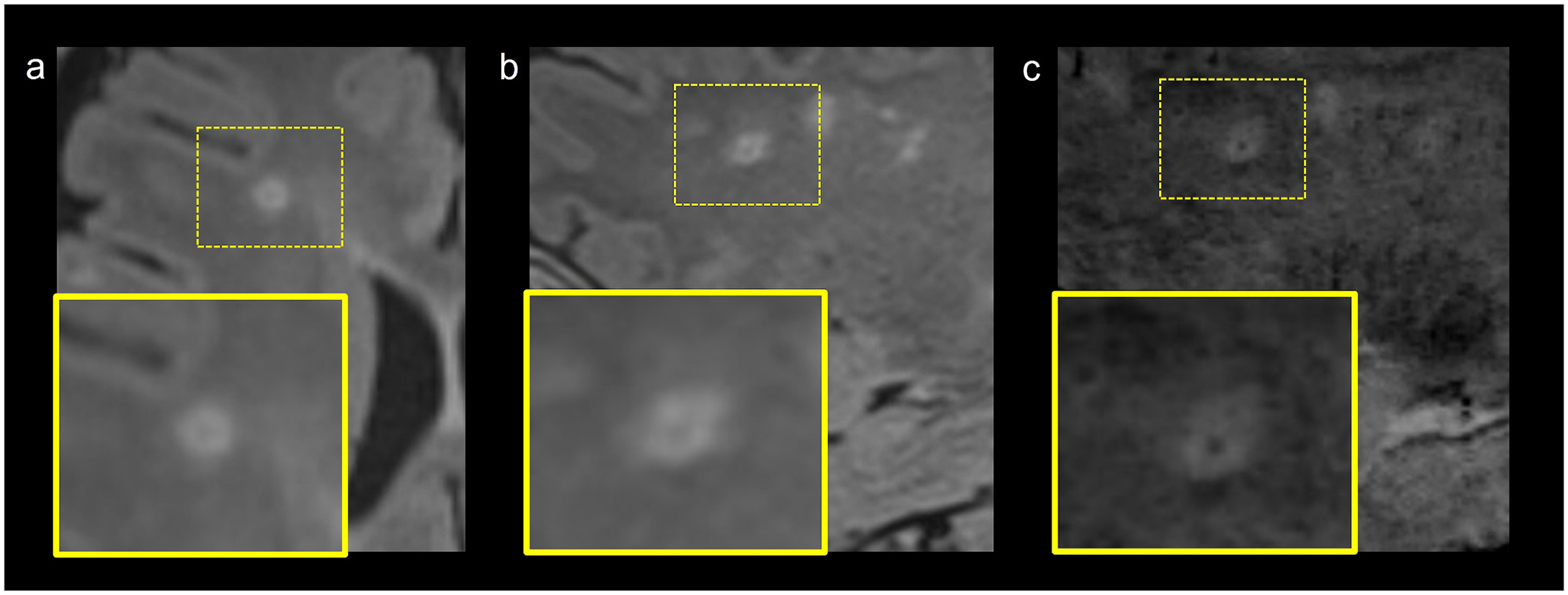
Scan from an individual with known diagnosis of multiple sclerosis obtained as part of routine clinical practice (Siemens Skyra 3 T). Axial (a) and sagittal (b) FLAIR showed a hyperintense lesion with a central hypointensity, with corresponding sagittal SWI (c) demonstrating the same.

**Fig. 3.**
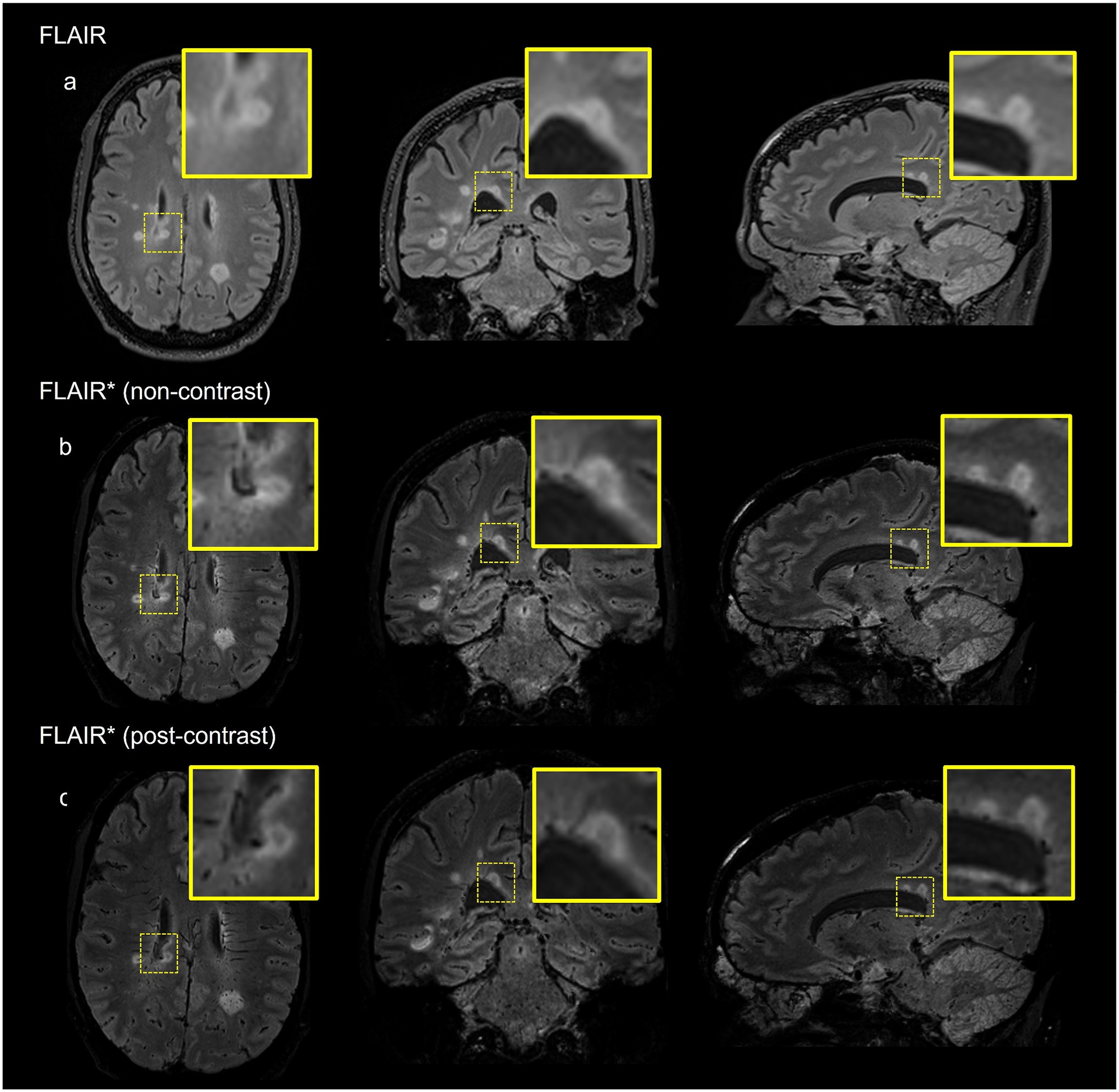
Example of a central FLAIR hypointensity in three planes (a), consistent with CVS on non-contrast (b) and post-contrast FLAIR* (c).

**Fig. 4.**
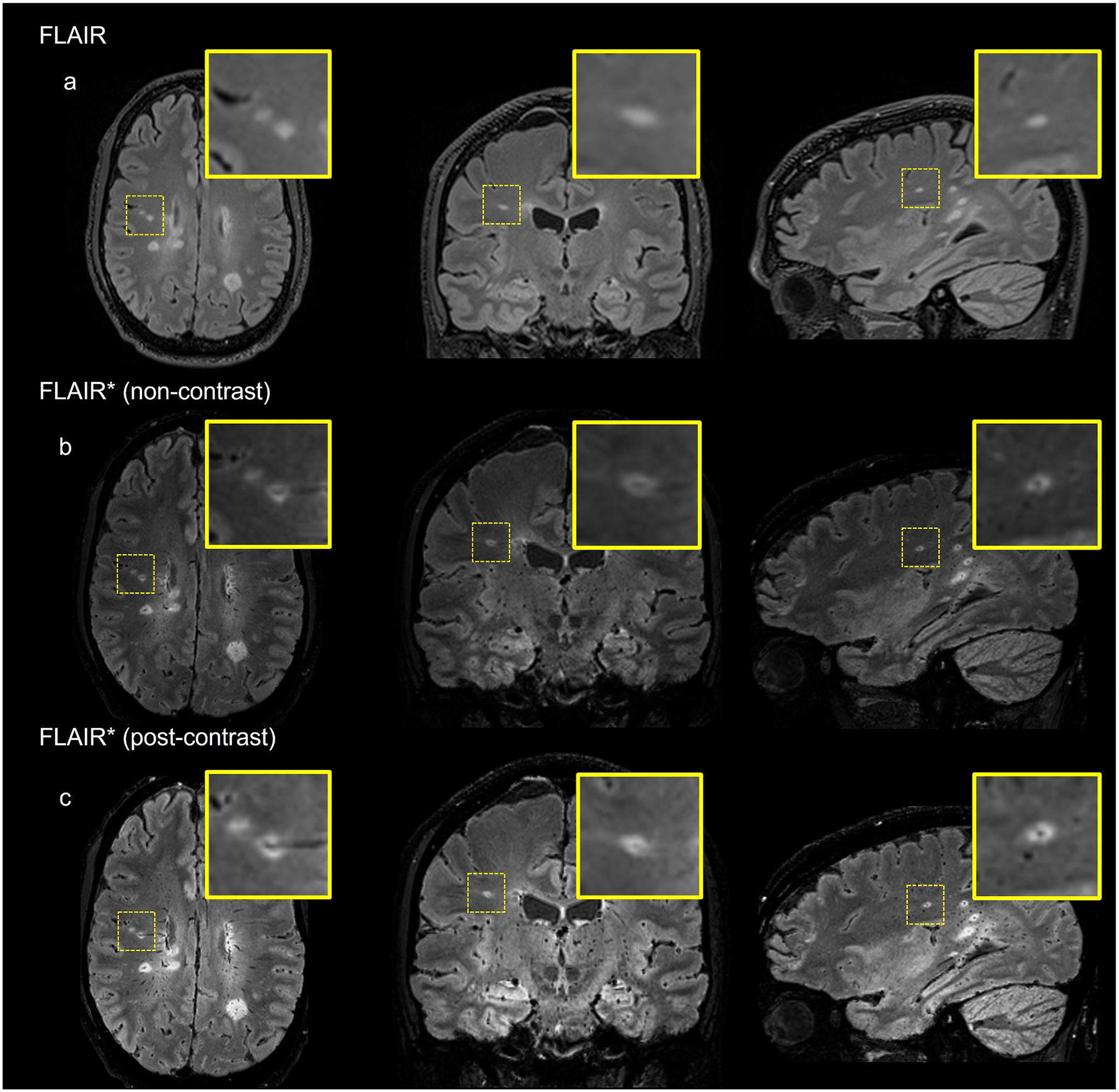
Example of a FLAIR hyperintense lesion without a central hypointensity (a), but a clear CVS on non-contrast (b) and post-contrast FLAIR* (c).

Initial assessment of lesions for CVS was conducted on post-contrast FLAIR* and performed by a trained rater (LD), blinded to diagnosis (Fig. 1). Lesions were marked as CVS+, CVS-, or excluded, according to NAIMS criteria for CVS assessment. Lesions were categorized at the time of initial assessment (by LD) as deep white matter (DWM), periventricular (PV), juxtacortical (JC), or infratentorial (IT), and which was not re-assessed during subsequent rating. Lesion coordinates were saved from this initial rating and were used for lesion rating via ITK SNAP software for the second rating on native FLAIR alone by a second blinded and trained rater (KT) [14]. Central FLAIR hypointensity (Fig. 2), was considered present if meeting NAIMS criteria for CVS, otherwise absent. A subset of scans was previously rated on non-contrast FLAIR* (n= 38, 3 sites with Siemens and 2 with Philips scanner) by the same initial rater (LD), and those lesion ratings were compared to coordinate-matched FLAIR ratings (Figs. 3 and 4).

The first analysis assessed the performance of central FLAIR hypointensity to post-contrast FLAIR* for lesion CVS status. This included determining accuracy, sensitivity, and specificity at the lesion-level (total and stratified per topography), as well as for status of Select 3 (≥3 central hypointense lesions), Select 6 (≥6 central hypointense lesions), and ≥ 40% CVS + threshold, respectively. The second analysis assessed the performance of central FLAIR hypointensity to non-contrast FLAIR* as the standard, with same lesion-level approaches and CVS methods as in the first analysis. The third analysis assessed the diagnostic performance of Select 3, Select 6, and ≥ 40% CVS + threshold on FLAIR in comparison to non-contrast and post-contrast FLAIR* in those with scans rated across all three approaches and with an adjudicated diagnosis of MS or an alternative condition (n= 32). For the third analysis, participants with clinically or radiologically isolated syndrome were excluded to conservatively optimize inference about diagnostic utility in a cross-sectional cohort.

### 2.5. Statistical analysis

Contingency tables were based on rating results. Sensitivity, specificity, positive (PPV) and negative predictive value (NPV) were calculated and reported with a 95% confidence interval (CI). Accuracy was reported as percentage of concordance. Comparisons of results for MS diagnostic performance were performed with McNemar’s test. R software (version 4.4.2) was used for analysis.

## 3. Results

### 3.1. Comparison of central FLAIR hypointensity and FLAIR* CVS

The overall accuracy of central FLAIR hypointensity (n= 92; 1737 lesions) was 64.4% using post-contrast FLAIR* CVS ratings as a standard (Table 1). Sensitivity was 33.5% (29.9 – 37.2), ranging from 27.9% for juxtacortical to 42.9% for infratentorial. Specificity was 83.2% (80.8 – 85.3), ranging from 63.6% for infratentorial lesions to 83.7% for deep white matter lesions.

The overall accuracy of central FLAIR hypointensity was 72.8% (n= 38; 768 lesions) using non-contrast FLAIR* CVS ratings as a standard (Table 2). Sensitivity was 39.8% (33.1 – 46.8), while specificity was 84.9% (81.6 – 87.7). Sensitivity ranged from 26.3% for juxtacortical to 75.0% for infratentorial. Specificity ranged from 79.0% for periventricular to 86.0% for juxtacortical.

### 3.2. Comparison of FLAIR and FLAIR* for CVS rating methods

Across 92 matched scans using post-contrast FLAIR* as the standard, FLAIR accuracy for Select 3 was 67.1%, for Select 6 was 77.3%, and 60.2% for the 40% CVS + threshold (Table 1). Across 38 matched scans using non-contrast FLAIR* as the standard, FLAIR accuracy for Select 3 and Select 6 was 76.3%, while 79% for the ≥ 40% CVS + threshold (Table 2).

### 3.3. Diagnostic performance of CVS rating methods on FLAIR and FLAIR* for MS

In 32 participants with an adjudicated diagnosis of MS or an alternative one, after exclusion of those with clinically or radiologically isolated syndrome, the diagnostic accuracy of Select 3 was 68.8% on post-contrast FLAIR*, 71.9% on non-contrast FLAIR*, and 50.0% on FLAIR (Table 3). For Select 3, sensitivities were similar across methods, but non-contrast FLAIR* had a higher specificity than FLAIR (68.4% vs 31.6%, p= 0.008). The accuracy of Select 6 for a diagnosis of MS was 84.4% for post-contrast FLAIR*, 81.3% for non-contrast FLAIR*, and 59.4% for FLAIR. Although sensitivities were similar when applying Select 6, post-contrast FLAIR* showed the highest point estimate (76.9%). With Select 6, FLAIR had a lower specificity (63.2%) than post-contrast FLAIR* (98.5%, p= 0.035) or non-contrast FLAIR* (100.0%, p= 0.008). As an approach in accordance with the 2024 McDonald criteria for CVS + status, 3/10 of those with MS and scan positive for post-contrast FLAIR* Select 6 were missed with the FLAIR approach. Conversely, 5/7 of those with non-MS diagnoses and scans positive for FLAIR Select 6 were not confirmed with post-contrast FLAIR*. Based on ≥ 40% CVS + threshold, the diagnostic accuracy of FLAIR was 59.4%, while 84.4% for post-contrast FLAIR* and 75.0% for non-contrast FLAIR*. For the same threshold, the sensitivity of FLAIR (15.4%) was lower than of post-contrast FLAIR* (84.6%, p= 0.003) or of non-contrast FLAIR* (53.9%, p= 0.025), while specificities were similar (84.2% – 89.5%).

## 4. Discussion

Results of our study demonstrated that FLAIR images alone are not reliable for assessment of CVS status and accurate MS diagnosis in a population referred for initial diagnostic assessment. Regarding lesion ratings, sensitivity was lower than specificity across defined topographies, respectively, though estimates may have been influenced by relative availability of eligible lesions; with deep white matter as most common and infratentorial as least common. Although the overall accuracy of central FLAIR hypointensity at the lesion level approached 65% in comparison to post-contrast FLAIR* CVS, and 73% in comparison to non-contrast FLAIR* CVS, the diagnostic performance of FLAIR Select 3, Select 6, or ≥ 40% CVS + threshold was close to equal chances (50–60%). From the latter group, Select 3 had poor specificity, while ≥ 40% CVS + threshold demonstrated a notably low sensitivity. This underscores the need for susceptibility-sensitive MRI sequences for the assessment and application of CVS, as is emphasized by NAIMS consensus [2].

With the inclusion of CVS into the MS diagnostic criteria algorithm, accessibility and familiarity with appropriate techniques is essential. Although CVS is not necessary for a diagnosis of MS, it may offer an earlier and accurate diagnosis when applied within the recommended diagnostic framework in some patients [8]. Current work supports the ongoing implementation efforts for CVS, including updates to institutional MRI protocols for demyelinating disease, which include a susceptibility-sensitive sequence to enable detection of CVS [15]. In addition to FLAIR* as a dedicated sequence, combinations of FLAIR with susceptibility-weighted imaging or T2*-weighted sequences may provide adequate visualization to reliably detect CVS, with increased detection rates by use of higher fields or intravenous gadolinium-based contrast [15].

**Fig. 5.**
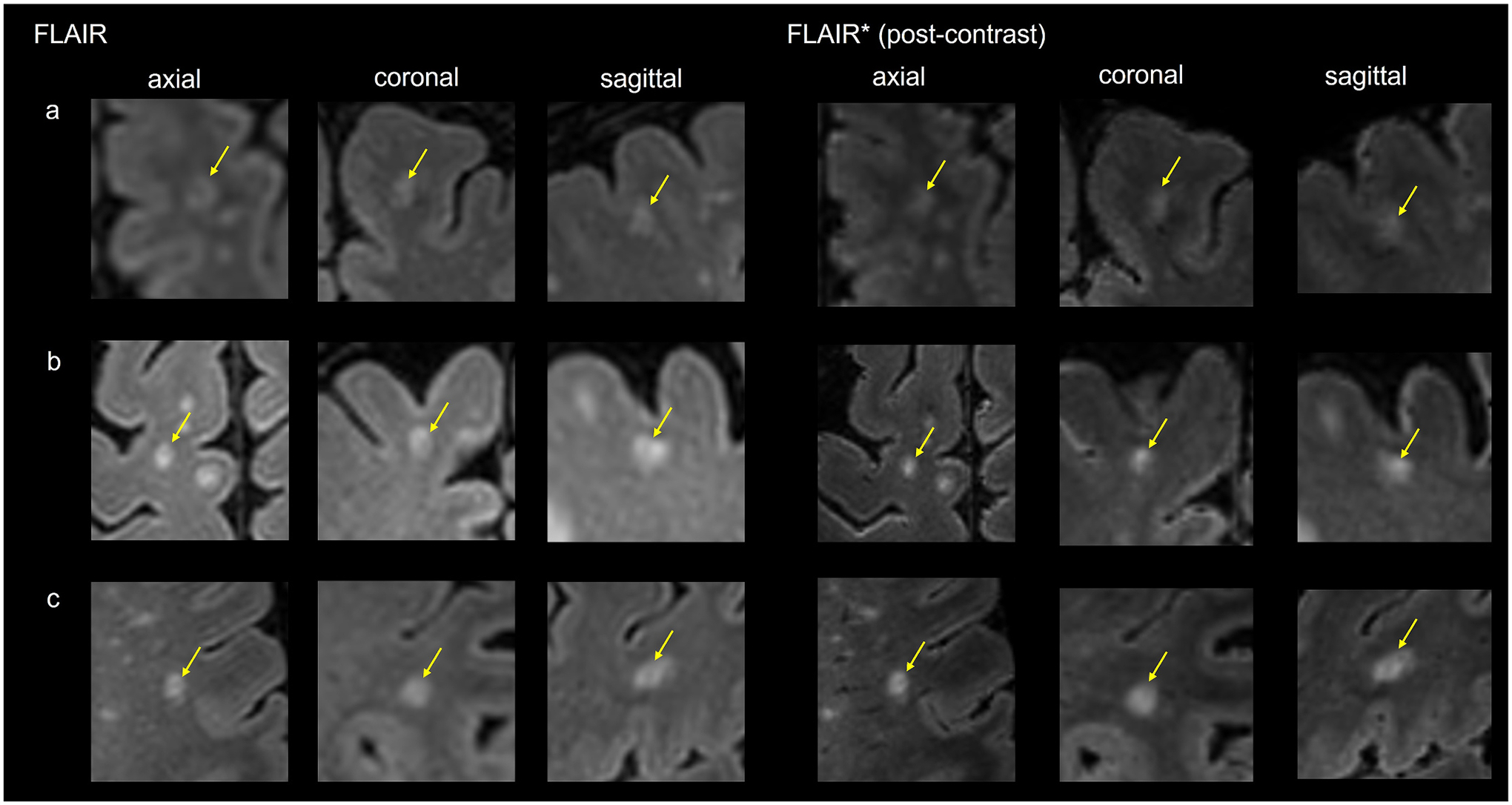
Examples of false positive findings on FLAIR, as characteristic central hypointensity was absent on post-contrast FLAIR* (a,b), or the lesion demonstrated multiple peripheral veins which were not evident on FLAIR (c).

Conversely, detection of central FLAIR hypointensity may not represent a true CVS, as evidenced by the relatively modest specificity found in this study, and such images should not be used for CVS application. Although FLAIR is not optimized to reliably visualize vascular structures, with increased resolution and refinement of FLAIR imaging it is plausible that some larger veins are detected as hypointensity, but especially problematic may be the CVS-like findings demonstrating faint conspicuities resembling a possible vein or when additional veins within a lesion are not detected (Fig. 5). The finding of central FLAIR hypointensity will likely depend on the imaging protocol, when the center of the lesion is closer to hydrogen content of the water, but may also represent an enlarged perivascular space, central cavitation within a lesion, or central gliosis in destructive lesions. Further histopathological investigations may uncover the causes of false positive FLAIR findings, while future high-resolution sequences may improve accurate detection. Despite ever-growing reliance on neuroimaging to improve the diagnostic process in MS, misinterpretation of findings may lead to misdiagnosis [16]. The same concern may exist for CVS as one of the new additions within McDonald 2024 revisions, but radiological definitions of CVS and associated recommendation provided by NAIMS should be strictly followed to ensure appropriate application [2].

Limitations include incomplete matching of imaging data, use of single rater with variable experience between individual raters, and lack of longitudinal follow-up for participants for diagnostic purposes. Previous assessment of inter-rater agreement for CVS based on the same imaging data showed substantial agreement for lesion rating on non-contrast (88%, κ= 0.71) and post-contrast (87%, κ= 0.73) FLAIR* [4]. Strengths of this study include a multi-center sample with different scanners, trained raters, and quality imaging data with a pre-specified protocol. The current study was based exclusively on 3-tesla MRI, but scans obtained on 1.5-tesla may provide adequate resolution for CVS assessment with FLAIR* [17]. As previously demonstrated, and recapitulated in this study, addition of contrast increases diagnostic sensitivity of FLAIR* CVS methods [4]. The larger CAVS-MS (n = 400), initiated after this pilot, may serve as a resource for further assessment and validation of current findings [18].

## 5. Conclusions

Assessment for CVS should be conducted according to NAIMS consensus recommendations, with a susceptibility-sensitive sequence as part of the imaging protocol for such purposes. Although FLAIR images may demonstrate a central hypointensity within a lesion, the sequence alone cannot be used to reliably rate CVS. Greater availability and familiarity with CVS as one of the newly included biomarkers in the MS diagnostic criteria will be critical to minimize misinterpretation of imaging findings.

## Supplementary Material

1

## Figures and Tables

**Table 1:** Results of lesion ratings across topographies and for abbreviated CVS methods based on FLAIR, with post-contrast FLAIR* as the standard (92 participants; 1737 lesions). Results are reported with 95% confidence intervals.

	Accuracy (%)	Sensitivity (%)	Specificity (%)	True positive (n)	False positive (n)
All lesions (n= 1737)	64.4	33.5 (29.9 – 37.2)	83.2 (80.8 – 85.3)	220	182
Deep white matter (n= 1402)	67.9	33.4 (29.0 – 38.0)	83.7 (81.2 – 86.0)	147	157
Periventricular (n= 136)	44.9	35.9 (26.7 – 46.0)	72.7 (54.5 – 86.7)	37	9
Juxtacortical (n= 160)	53.8	27.9 (18.8 – 38.6)	83.8 (73.4 – 91.3)	24	12
Infratentorial (n= 39)	48.7	42.9 (24.5 – 62.8)	63.6 (30.8 – 89.1)	12	4
Select 3 (n= 92)	67.1	48.5 (30.8 – 66.5)	78.2 (65.0 – 88.2)	42	8
Select 6 (n= 92)	77.3	77.8 (64.4 – 88.0)	76.5 (58.8 – 89.3)	16	12
≥40% CVS+ (n= 92)	60.2	31.7 (18.1 – 48.1)	85.1 (71.7 – 93.8)	13	7

**Table 2:** Results of lesion ratings across topographies and for abbreviated CVS methods based on FLAIR, with non-contrast FLAIR* as the standard (38 participants; 768 lesions). Results are reported with 95% confidence intervals.

	Accuracy (%)	Sensitivity (%)	Specificity (%)	True positive (n)	False positive (n)
All lesions (n= 768)	72.8	39.8 (33.1 – 46.8)	84.9 (81.6 – 87.7)	82	85
Deep white matter (n= 633)	75.0	41.8 (36.7 – 50.2)	85.0 (81.5 – 88.1)	61	73
Periventricular (n= 56)	50.0	35.1 (20.2 – 52.5)	79.0 (54.4 – 94.0)	13	4
Juxtacortical (n= 69)	69.6	26.3 (9.2 – 51.2)	86.0 (73.3 – 94.2)	5	7
Infratentorial (n= 10)	80.0	75.0 (19.4 – 99.4)	83.3 (35.9 – 99.6)	3	1
Select 3 (n= 38)	76.3	94.4 (72.7 – 99.9)	60.0 (36.1 – 80.9)	17	8
Select 6 (n= 38)	76.3	100.0 (59.0 – 100.0)	71.0 (52.0 – 85.8)	7	9
≥40% CVS+ (n= 38)	79.0	40.0 (12.2 – 73.8)	92.9 (76.5 – 99.1)	4	2

**Table 3:** Performance of abbreviated and percentage-based CVS methods for diagnosis of multiple sclerosis based on 32 participants (13 with multiple sclerosis), with comparisons between FLAIR and FLAIR* approaches.

Method	Accuracy (%)	Sensitivity (%)	Specificity (%)	Positive predictive value (%)	Negative predictive value (%)
**Select 3**					
Post-contrast FLAIR*	68.8	100.0 (75.3 – 100.0)	47.4 (24.5 – 71.1)	56.5 (45.9 – 66.6)	100.0 (66.4 – 100.0)
Non-contrast FLAIR*	71.9	76.9 (46.2 – 95.0)	68.4 (43.5 – 87.4)	62.5 (44.7 – 77.5)	81.3 (60.6 – 92.5)
FLAIR	50.0	76.9 (46.2 – 95.0)	31.6 (12.6 – 56.6)	43.5 (33.4 – 54.1)	66.7 (37.8 – 86.8)
p-values					
Post-contrast FLAIR* vs. FLAIR		0.083	0.083	0.014	0.042
Non-contrast FLAIR* vs. FLAIR		–	0.008	0.007	0.070
**Select 6**					
Post-contrast FLAIR*	84.4	76.9 (46.2 – 95.0)	89.5 (66.9 – 98.7)	83.3 (56.6 – 95.0)	85.0 (67.5 – 93.9)
Non-contrast FLAIR*	81.3	53.9 (25.1 – 80.8)	100. (82.4 – 100.0)	100.0 (59.0 – 100.0)	76.0 (63.8 – 85.1)
FLAIR	59.4	53.9 (25.1 – 80.8)	63.2 (38.4 – 80.8)	50.0 (31.6 – 68.5)	66.7 (50.3 – 79.8)
p-values					
Post-contrast FLAIR* vs. FLAIR		0.083	0.035	0.004	0.020
Non-contrast FLAIR* vs. FLAIR		–	0.008	<0.001	0.030
**≥40% CVS + threshold**					
Post-contrast FLAIR*	84.4	84.6 (54.6 – 98.1)	84.2 (60.4 – 96.6)	78.6 (55.9 – 91.4)	88.9 (68.8 – 96.7)
Non-contrast FLAIR*	75.0	53.9 (25.1 – 80.8)	89.5 (66.7 – 98.7)	77.8 (46.2 – 93.4)	73.9 (60.7 – 83.9)
FLAIR	59.4	15.4 (1.9 – 45.5)	89.5 (66.7 – 98.7)	50.0 (13.8 – 86.2)	60.7 (53.9 – 67.1)
p-values					
Post-contrast FLAIR* vs. FLAIR		0.003	0.317	0.175	<0.001
Non-contrast FLAIR* vs. FLAIR		0.025	–	0.097	0.018

## Appendix A. Supplementary data

Supplementary data to this article can be found online at https://doi.org/10.1016/j.ejrad.2026.112707.
