# Supplementary material for: Comparison of central FLAIR hypointensity and central vein sign on FLAIR* in a diagnostic cohort
Source: Eur J Radiol. Author manuscript; Available in PMC 2026 Jul 22. (PMC13390084; doi:10.1016/j.ejrad.2026.112707)
Supplement: 1 [file NIHMS2190198-supplement-1.docx]

**Supplementary table 1.** Sequences obtained as part of study imaging protocol (all sequences comprised a 3D sagittal acquisition of entire brain).

| Sequence | Isotropic resolution (mm) | TR/TE | Acquisition time | Other parameters |
| --- | --- | --- | --- | --- |
| Non-contrast T2-weigthed 3D FLAIR | 1.0 | 4800/352 | 7 min 9s | Inversion time (TI) = 1800 ms |
| Non-contrast and post-contrast T2*-weighted 3D segmented echo-planar imaging (EPI) | 0.65 | 64/35 | 5 min 48 s | Flip angle = 10°,  EPI factor = 15 |
| Non-contrast and post-contrast T1-weighted 3D gradient-echo imaging | 1.0 | 7.8/3.0 | 3 min 16 s | Flip angle= 18° |
